# What is the role of leisure-time physical activity in the association between neighborhood environmental characteristics and hypertension in older adults? The EpiFloripa Aging Cohort study

**DOI:** 10.1016/j.pmedr.2024.102909

**Published:** 2024-10-18

**Authors:** Viviane Nogueira de Zorzi, Francisco Timbó de Paiva Neto, Thamara Hubbler Figueiró, Danielle de Amaral Macedo, Lucas Gomes Alves, Willen Remon Tozetto, Eleonora d’Orsi, Cassiano Ricardo Rech

**Affiliations:** a Postgraduation Program in Physical Education, Federal University of Santa Catarina, R. Eng. Agronômico Andrei Cristian Ferreira, s/n – 88040-900, Florianópolis, Brazil; b Hospital Israelita Albert Einstein. Av. Albert Einstein, 627/701 - Morumbi, São Paulo SP, 05652-900, Brazil; c Postgraduation Program in Public Health, Federal University of Santa Catarina, R. Eng. Agronômico Andrei Cristian Ferreira, s/n – 88040-900, Florianópolis, Brazil

**Keywords:** Hypertension, Blood Pressure, Aging, Walking, Built environment, Environment design, Physical activity

## Abstract

**Background:**

Research suggests a link between the urban built environment and blood pressure, potentially mediated by physical activity. This study aims to investigate the relationship between perceived neighborhood characteristics and blood pressure in older adults, as well as the mediating role of walking in this relationship.

**Methods:**

Data from the third wave of the EpiFloripa Aging Cohort Study in Florianopolis, Brazil (2017–2019; n = 1335) were used for this cross-sectional analysis. Blood pressure was measured using digital devices, and hypertension diagnosis relied on healthcare professionals’ information. The neighborhood environment was assessed with the Abbreviated Neighborhood Environment Walkability Scale, while walking was evaluated with the International Physical Activity Questionnaire. Multilevel logistic regression analyzed the association between the neighborhood environment, blood pressure, and hypertension. Structural equation modeling assessed the mediation effect of walking.

**Results:**

Hypertension prevalence was 85.3 %, with 69.0 % having objectively measured elevated blood pressure. Results showed that older adults perceiving better infrastructure for physical activity (OR: 0.88; CI: 0.78–0.99), increased safety in their neighborhood (OR: 0.88; CI: 0.79–0.99), traffic security (OR: 0.88; CI: 0.78–0.99) and better overall perception of the environment (OR: 0.73; IC: 0.55–0.98) had a lower likelihood of self-reported hypertension. The association between self-reported hypertension and overall perception of the environment was partially explained by leisure-time walking (β = -0.01; p < 0.05).

**Conclusion:**

Our findings suggest that public policies promoting the establishment of safe and supportive spaces for physical activity emerge as essential measures in the prevention and management of hypertension in older adults.

## Highlights

• Walking infrastructure associated with lower self-reported hypertension in elderly.
• Safety related to traffic and crime linked to lower self-reported hypertension.
• Better environmental perception lowers likelihood of self-reported hypertension.
• Walking have a mediating effect entre hypertension and environmental perception.
• Supportive environments for physical activity help prevent hypertension in elderly.

## 1 Introduction

Hypertension increases the risk of cardiovascular disease and stands as a primary contributor to premature mortality (Brouwers et al., 2021, Dzau and Hodgkinson, 2024, Zhou et al., 2017). The population impact of high blood pressure (BP) is increasing due to longer life expectancy, as the prevalence of hypertension is widely recognized to rise with age (Brouwers et al., 2021, Olsen et al., 2016). The prevalence of hypertension is higher in low- and middle-income countries, comprising 82 % of the global hypertensive population (Zhou et al., 2021). These regions also carry a significant and increasing burden of hypertension and related cardiovascular diseases, resulting in a considerable impact on the healthcare system of these countries (Miranda et al., 2019). In Brazil, approximately 65.1 % of the population aged 65 and older reported a diagnosis of hypertension. (Ministério da Saúde, 2023).

Some studies have indicated a potential connection between characteristics of the urban built environment and blood pressure (Avila-Palencia et al., 2022, Malambo et al., 2016a). Residing in neighborhoods characterized by higher walkability has been correlated with reduced blood pressure levels or a lower incidence of hypertension (Chiu et al., 2016; Chandrabose et al., 2019a). However, studies about the impact of the neighborhood environment on the blood pressure of older adults in low- and middle-income countries are scarce with mostly of the investigations predominantly conducted in Europe and North America (Avila-Palencia et al., 2022). Latin America, marked by significant urbanization levels and a diverse array of urban landscapes (Anza-Ramirez et al., 2022), remains underemphasized in this field of study. Thus, most studies establish a correlation between objective urban environmental metrics and blood pressure. However, only 5.6 % of these studies examine blood pressure in relation to the perceived neighborhood environment (Malambo et al., 2016a).

One strategy in the Action Plan for the Prevention and Control of Noncommunicable Diseases to reduce the incidence of arterial hypertension is to decrease physical inactivity, as it is one of the main risk factors. (WHO, 2018). A review provided compelling evidence that physical activity lowers blood pressure, highlighting an inverse relationship between physical activity and the onset of hypertension (Pescatello et al., 2019). Additionally, engagement in physical activity has been linked to a reduced risk of hypertension in older adults (Jia et al., 2018) and the walking, frequently cited as the most commonly engaged type of physical activity, is also widely acknowledged for its cardiovascular benefits (Ungvari et al., 2023).

Evidence shows that specific elements of the built environment provide opportunities for physical activity, particularly walking (Sallis et al., 2020, Tuckett et al., 2018, Van Cauwenberg et al., 2018, Zhang et al., 2024). Positive factors associated with older adults engaging in physical activity include walkability, pedestrian-friendly features, general safety, availability and accessibility of shops/commercial services and parks/open spaces (Sallis et al., 2020, Zhang et al., 2024). We hypothesized that the neighborhood environment serves as exposure influencing people’s behaviors, encouraging the practice of physical activity. Consequently, a greater satisfaction with the neighborhood environment may be associated with a decreased likelihood of high blood pressure and the diagnosis of hypertension. Therefore, this study aimed to investigate the association between neighborhood environmental characteristics and self-reported hypertension and measured BP in Brazilian older adults. Additionally, we examine the mediating role of leisure walking in this relationship.

## 2 Materials and Methods

### 2.1 Study design and ethical procedures

This cross-sectional analysis was conducted using data from the third wave of the longitudinal study: “Conditions of Health in Older Adults in Florianopolis − EpiFloripa Aging Cohort Study” which took place between October 2017 and December 2019. The city of Florianopolis had a population of 537,211 residents and a territorial area of 674,844 km^2^ according to the 2022 census by the Brazilian Institute of Geography and Statistics (IBGE, 2022). Additionally, the city features a Municipal Human Development Index (HDIM) of 0.847, ranking third among Brazilian municipalities (UNDP, 2013). The EpiFloripa Aging Cohort Study was conducted in accordance with the Declaration of Helsinki. The project obtained approval from the Ethics Research Committee with Human Subjects under number 1,957,977 (September 12, 2013; CAAE No. 16731313.0.0000.0121). After the explanation of the study objectives and collection procedures, all participants voluntarily agreed to participate in the study and signed the informed consent form.

### 2.2 Population sample and data collection

This study is a population-based, home-based, longitudinal cohort study. The baseline sampling method in 2009/10 employed a two-stage cluster approach, initially selecting census tracts and subsequently households. From 420 urban census tracts in Florianópolis (IBGE, 2000), 80 were systematically chosen based on the average monthly income of household heads (R$314.76 to R$5,057.77). Census tracts with fewer than 150 households were combined, and those with more than 500 were subdivided, resulting in 83 census tracts for data collection (Schneider et al., 2017). The sample size was determined by considering maximum variability (50 % prevalence), a four percent margin of error, and a 95 % confidence interval. Earlier publications provide comprehensive information on the study sample and methodology (Confortin et al., 2019, Schneider et al., 2017). Inclusion criteria at baseline (2009–2010) comprised men and women aged ≥ 60 years residing in an urban area in southern Brazil (Florianopolis/Santa Catarina). Exclusion criteria included individuals living in long-term care institutions, hospitals, and prisons. In summary, trained interviewers conducted face-to-face home interviews using a structured questionnaire, with data recorded on a netbook. Quality control was applied to 10 % of randomly selected interviews (Schneider et al., 2017).

The third wave of the study included participants who had been followed since the baseline in 2009/2010, as well as new older adults added to refresh the sample, resulting in a total of 1,335 older adults (Confortin et al., 2022). The cross-sectional analysis was chosen due to challenges in maintaining longitudinal follow-up with an aging population and the need for complete data on various exposures and outcomes, which could compromise sample size and statistical power if approached longitudinally.

### 2.3 Outcomes

We employed two distinct health outcome measures: a) objectively measured systolic and diastolic blood pressure and b) self-reported hypertension diagnosis. Systolic and diastolic blood pressure were measured twice during the interview process – initially at the beginning of the interview and then approximately 40 min later to mitigate the impact of any preceding activity. Participants were instructed not to consume coffee, chimarrão, tea, or any other food or beverage that could influence their blood pressure before or during the interview. Blood pressure measurements were taken with the participant seated, with their arm supported at a 90-degree angle on a table.The measurements were taken on both arms. The mean of the two measurements was subsequently calculated and adopted. Digitally read devices, specifically the Techline Z-40 model (Techline, Taiwan, China), calibrated for research purposes, were utilized. Participants were categorized into two groups: normal blood pressure (<130/90 mmHg) and high blood pressure (≥130/90 mmHg) (Barroso et al., 2021). For the analysis of the self-reported diagnosis of hypertension, participants answered the following question: Has any physician or healthcare professional ever told you that you have/had hypertension (high blood pressure)? The responses were dichotomous: Yes/No.

### 2.4 Exposures

The perceived neighborhood environment was evaluated through the instrument adapted from the international Neighborhood Environment Walkability Scale Abbreviated (A-NEWS), translated and validated in Brazil (Florindo et al., 2012, de Malavasi and M., Duarte, M. de F. da S., Both, J., Reis, R.S., 2007, Saelens et al., 2003). The adapted instrument consists of 20 questions that measure the perception of the respondents about the neighborhood environment characteristics. These questions form the following domains: land use mix, infrastructure for walking/cycling, safety related from traffic and safety related from crime. Participants were inquired about their perception of places they can walk to within a 15-minute distance in their neighborhood environment. The responses could be either “yes” (1) or “no” (0), forming scores for each block, which were subsequently standardized into z-scores. Additionally, we created a variable representing the overall environment perception by summing the scores from all domains of this instrument. For analytical purposes, the tertile values for overall environmental perception were assessed and classified as low, intermediate, and high according to Table 1.

**Table 1:** Characteristics of Brazilian older adults of the EpiFloripa Aging Cohort Study. Florianopolis, Brazil, 2017–2019 (n = 1,335).

Variables	n	%(CI_95%_)
Sex		
Female	814	63.0 (59.8–66.2)
Male	521	37.0 (33.8–40.2)

Age group (in years)		
60 to 69	461	30.2 (25.4–35.6)
70 to 79	554	43.7 (39.2–48.3)
≥80	320	26.1 (22.4–30.3)

Education level (in years)		
≤4 years	445	32.2 (26.8–38.3)
5 – 11 years	454	34.3 (29.6–39.3)
≥12 years	431	33.5 (27.1–40.6)

Household Income (BMW^a^)		
≤ 1 MW	140	9.8 (7.86–12.1)
> 1 MW ≤ 3 MW	367	28.3 (23.4–33.8)
> 3 MW ≤ 5 MW	247	18.9 (16.4–21.6)
> 5 MW ≤ 10 MW	327	23.6 (19.9–27.8)
> 10 MW	238	19.4 (15.6–23.4)

Smoking habit		
No	784	59.8 (56.1–63.4)
Former Smoker	429	30.9 (27.8–34.3)
Smoker	122	9.3 (7.4–11.5)

Body Mass Index^b^		
Underweight	118	8.8 (7.2–10.8)
Eutrophic	445	33.8 (30.5–37.3)
Overweight	694	57.4 (53.9–60.7)

Waist circunferenc (WC^c^)		
Normal	520	41.2 (38.1–44.4)
Increased	732	58.8 (55.6––61.9)

Physical Activity levels^d^		
Insufficiently active	953	72.2 (68.5–75.6)
Active	359	27.8 (24.4–31.5)

Hypertension^e^		
Yes	819	59.5 (54.5–64.3)
No	516	40.5 (35.7–45.5)

Blood Pressure^f^		
Normal	409	31.0 (27.1–35.1)
Increased	916	69.0 (64.9–72.9)

Antihypertensive medication use		
Yes	907	67.9 (65.4–70.4)
No	428	32.1 (29.6–34.6)

Total Environment Perception in tertiles^g^		
Low	568	42.5 (39.9–45.2)
Intermediate	464	34.8 (32.2–37.3)
High	303	22.7 (20.5–25.0)
	**Median**	**IQR^h^**
Land use mix-diversity^g^	0.12	−0.51–0.75
Infrastructure for walking/cicling^g^	0.32	−0.36–0.99
Saffety related from traffic^g^	0.30	−0.57–1.17
Saffety related from crime^g^	0.14	−0.80–1.08

^a^ BMW = Brazilian minimum wage; ^b^BMI Cutoff point (Lipschitz, 1994) (< 22 Kg/m^2^, underweight ≥ 22 e < 27 Kg/m2: eutrophic; ≥27 Kg/m^2^: overweight); ^c^WC = Cutoff point (WHO, 1995) women > 80 cm, male > 94 cm; ^d^Insufficiently active (<150 min/week), Active (>150 min/week); ^e^reported having hypertension diagnosed by a physician;); ^f^Blood Pressure cutoff point (Barroso et al., 2021) ≥ 130/90; ^g^ = variables presented in z scores; ^h^IQR = Interquartile Range (percentil 25 and percentil 75).

### 2.5 Mediator variables

Leisure-time walking were assessed using the validated extended version of the International Physical Activity Questionnaire (IPAQ). This version is designed specifically for older individuals in Brazil (Benedetti et al., 2004). The weekly frequency and duration in minutes of leisure walking were multiplied to calculate the total minutes of activity performed during the week. The variable was used in continuous form.

### 2.6 Covariates

A theoretical model was elucidated through the construction of a Directed Acyclic Graph (DAG). This graphical representation created using DAGitty software (version 3.0; Nijmegen, GE, The Netherlands) (Textor et al., 2016) aimed to illustrate the interplay of covariates in the association between neighborhood environment perception and blood pressure (see Appendix A). The DAG construction followed graphical criteria, employing adjusted covariates to establish a minimized set and mitigate variable selection bias (Herńan and Robins, 2012).

The DAG-guided selection of covariates included variables such as sex (female and male), age (“60–69 years old”, “70–79 years old” and “80 years old or more”), education (years of study categorized as “≤4 years,” “5–11 years,” and “≥12 years”), and per capita family income measured in minimal wages (grouped into categories: ≤1; > 1 and ≤ 3; > 3 and ≤ 5; > 5 and ≤ 10; > 10 minimal wages). The income categories were determined based on the Brazilian minimum wage value in 2017 (R$ 937.00).

Information on behavior-modifying factors, such as smoking habits (no/former smoker/smoker), and health status indicators like body mass index, categorized as underweight (<22 kg/m2), eutrophic (22–27 kg/m2), and overweight (>27 kg/m2) (Lipschitz, 1994) as well as high waist circumference (cutoff point for women > 80 cm and for male > 94 cm) (WHO, 1995) are also provided. The participants' census tract was used as a contextual variable in the multilevel analysis, which is important for examining neighborhood-level influences.

### 2.7 Statistical analysis

Descriptive analysis of the data is presented in terms of absolute and relative frequencies, as well as the median, standard deviation (SD) and interquartile range (IQR). The minimum set of adjusted covariates, determined by applying the back-door criterion to the DAG, included sex, age, education, and family income (see Appendix A).

The relationship between neighborhood perception and blood pressure/hypertension was analyzed using Multilevel Logistic Regression. Two models were tested: the crude model and an adjusted model, as indicated by the DAG. The analysis had two levels: participants (level 1) and census tracts (level 2), where the census tract served as a contextual variable. This allowed for the examination of neighborhood-level influences on blood pressure and hypertension by accounting for shared characteristics within census tracts. The models were evaluated using Akaike Information Criterion (AIC) and Bayesian Information Criterion (BIC). The intraclass correlation coefficient (ICC) was calculated to assess variance attributed to census tracts (Merlo et al., 2005). Odds ratios (OR) and 95 % confidence intervals (CI95%) were provided for each model.

Structural Equation Modeling (SEM) was employed to examine the total, direct, and indirect effects (Richiardi et al., 2013) of overall environment perception on hypertension, along with the mediating role of walking leisure. The robust weighted least squares mean and variance adjusted estimator (WLSMV) were used to control for differences in residual variances. Modification indices (MI) helped identify additional paths to improve model fit, with new models constructed if MI values exceeded 10 and modifications were theoretically justified (Wang and Wang, 2019). To assess whether the model exhibited a good fit, the following fit indices were taken into account: p > 0.05 for the χ2 (Kline, 2016), p < 0.05 and an upper limit of the 90 % confidence interval < 0.08 for the root mean square error of approximation (Wang et. al., 2019), values > 0.90 for the comparative fit index and the Tucker Lewis index (Marôco, 2021), values < 0.05 for the standardized root mean square residual (Kline, 2016). The findings were presented following the guidelines outlined in A Guideline for Reporting Mediation Analyses (AGReMA) recommendations (Lee et al., 2021). The analysis was performed using Stata 14.0 software (StataCorp, College Station, TX, USA). SEM analysis was conducted using the “lavaan” package in R (Rosseel, 2011) and a p value < 0.05 was considered statistically significant for all analyses.

## 3 Results

### 3.1 Sociodemographic and health-related characteristics

A total of 1,335 older adults participated in the study, 63.0 % were women, most of the sample was between 70 and 79 years old (43.7 %), had between five and 11 years of education (34.3 %), and 90 % had an income higher than one minimum wage. In the sample, 59.8 % reported not smoking, 57.4 % were overweight, 58.8 % with waist circumference increased and 72.2 % do not meet the global recommended levels of physical activity. The prevalence of self-reported hypertension in sample was 59.5 % (CI95%: 54.5; 64.3) and objectively measured was 69.0 % (CI95%: 64.9; 72.9) (Table 1). The mean SBP and DBP was 139.9 (SD = 19.8) mmHg and 80.7 (SD = 12.6) mmHg, respectively (information not provided in the table).

### 3.2 Association between blood Pressure, hypertension and perceived environment characteristics

Table 2 shows the multilevel logistic regression results for the odds of presented high blood pressure. In both the crude and adjusted analyses, no significant associations were observed between perceived environmental features and the outcome. Adjusted analyses, which included the variables selected by the DAG as well as the variable for antihypertensive medication use, were conducted and are presented in the supplementary material (see Appendix B). In relation to high blood pressure, when compared to the null model, the intraclass correlation demonstrated a 0.3 % increase for Land Use Mix-Diversity, a 0.2 % increase for Safety Related to Crime, and a 0.4 % increase for Total Environment Perception. On the other hand, there was a 0.7 % reduction in the intraclass correlation for walking/cycling infrastructure.

**Table 2:** Effect of neighborhood environment perception on objectively measured blood pressure in older adults from Florianopolis, Brazil. EpiFloripa Aging Cohort Study, 2017–2019 (n = 1,305).

**Variables**	**High Blood Pressure**			
	OR Crude (CI_95%_)	p-value	OR Adjusted (CI_95%_)**^†^**	p-value
**Empty Model**				
	2.31(1.99–2.68)	0.001	−	−
AIC	1635.2			
BIC	1645.6			
ICC (%)	5.1			

**Land use mix-diversity**				
	1		1	
	1.10 (0.97–1.25)	0.13	1.09 (0.97–1.25)	0.15
AIC	1634.9		1598.6	
BIC	1650.5		1634.8	
ICC (%)	5.4		4.6	

**Infrastructure for walking/cycling**				
	1		1	
	0.91 (0.80–1.03)	0.15	0.91 (0.80–1.04)	0.17
AIC	1635.2		1598.8	
BIC	1650		1635.0	
ICC (%)	4.4		4.1	

**Safety related from traffic**				
	1		1	
	0.95 (0.85–1.08)	0.49	0.96 (0.85–1.09)	0.52
AIC	1636.7		1600.2	
BIC	1652.3		1636	
ICC (%)	5.1		4.6	

**Saffety related from crime**				
	1		1	
	1.03 (0.91–1.16)	0.64	1.01 (0.89–1.15)	0.84
AIC	1637.0		1600.6	
BIC	1652.6		1636.8	
ICC (%)	5.3		4.7	

**Total Environment Perception- tercile**				
Low	1		1	
Intermediate	1.14 (0.80–1.52)	0.36	1.15 (0.87–1.55)	0.31
High	1.08 (0.78–1.49)	0.63	1.07 (0.78–1.50)	0.64
AIC	1638.4		1601.6	
BIC	1659.1		1643.0	
ICC (%)	5.5		4.9	

AIC: Akaike Information Criterion; BIC: Bayesian Information Criterion; ICC: Intraclass Correlation; 95 % CI: 95 % Confidence Interval. †: Adjusted model with sex, age, education level and household income.

Note: Neighborhood Environment Perception variables are presented as z-score. The AIC and BIC values were smaller for the adjusted models, indicating a better fit compared to the unadjusted models.

Table 3 shows the results of association between self-reported hypertension and built environment. In the crude analysis, all independent variables exhibited a significant association with the outcome. After adjusting for confounding, the likelihood of reporting a diagnosis of hypertension was found to be lower among the older adults who perceived favorable infrastructure for walking/cycling (OR: 0.88; 95 %CI: 0.78–0.99; p = 0.03), more safety related to traffic (OR: 0.88; 95 %CI: 0.78–0.99; p = 0.02), more safety related to crime (OR: 0.88; 95 %CI: 0.79–0.99; p = 0.03), and among those who had a higher overall environmental perception (OR: 0.73; 95 %CI: 0.55–0.98; p = 0.03). Furthermore, it was noted that, for both outcomes, the Intraclass Correlation Coefficient (ICC) values exhibited minor fluctuations across the analyzed models. This indicates that the census tract variable contributed minimally to explaining the variance in outcomes across different levels of analysis (refer to Table 2, Table 3).

**Table 3:** Effect of neighborhood environment perception on self-reported hypertension in older adults from Florianopolis, Brazil. EpiFloripa Aging Cohort Study, 2017–2019 (n = 1,314).

**Variables**	**Hypertension**			
	OR crude (CI_95%_)	p-value	OR Adjusted(CI_95%_)**^†^**	p-value
**Empty Model**				
	1.59 (1.42–1.77)	0.001	−	−
AIC	1783.3			
BIC	1788.5			
ICC (%)	0.0			
**Land use mix-diversity**				
	1		1	
	**0.88 (0.79**–**0.99)**	**0.02**	0.97 (0.86–1.10)	0.64
AIC	1780.4		1696.9	
BIC	1790.8		1728.0	
ICC (%)	0.0		0.0	
**Infrastructure for walking/cycling**				
	1		1	
	**0.84 (0.75**–**0.94)**	**0.003**	**0.88 (0.78**–**0.99)**	**0.03**
AIC	1776.2		1692.5	
BIC	1786.6		1723.6	
ICC (%)	0.0		0.0	
**Safety related from traffic**				
	1		1	
	**0.87 (0.78**–**0.97)**	**0.01**	**0.88 (0.78**–**0.99)**	**0.02**
AIC	1779.4		1692.4	
BIC	1789.8		1723.5	
ICC (%)	0.0		0.0	
**Safety related from crime**				
	1		1	
	**0.88 (0.79**–**0.99)**	**0.02**	**0.88 (0.79**–**0.99)**	**0.03**
AIC	1780.5		1692.7	
BIC	1790.9		1723.8	
ICC (%)	0.0		0.0	
**Total Environment Perception − tercile**				
Low	1		1	
Intermediate	0.91 (0.71–1.18)	0.47	1.06 (0.81–1.38)	0.67
High	**0.65 (0.49**–**0.87)**	**0.003**	**0.73 (0.55**–**0.98)**	**0.03**
AIC	1778.3		1692.9	
BIC	1793.9		1729.2	
ICC (%)	0.0		0.0	

AIC: Akaike Information Criterion; BIC: Bayesian Information Criterion; ICC: Intraclass Correlation; 95 % CI: 95 % Confidence Interval. †: Adjusted model for sex, age, education level and household income.

Note: Neighborhood Environment Perception variables are presented as z-score. The AIC and BIC values were smaller for the adjusted models, indicating a better fit compared to the unadjusted models.

### 3.3 Indirect effect of walking in association between overall environment perception and hypertension

The simplified theoretical model is presented (see Appendix C) and the standardized coefficients of the direct, indirect, and total effects of overall environment perception on leisure-time walking and self-reported hypertension are presented in Table 4. Overall environment perception shows negative direct (βpad = -0.09; p < 0.01) and total effects (βpad = -0.21; p < 0.05) on self-reported hypertension. Although the sum of the direct and indirect effects results in a total effect of −0.01, when all pathways in the path model are considered, the total effect is −0.21. Engagement in leisure-time walking exerted a partial and statistically significant mediating effect on the relationship between overall environmental perception and self-reported hypertension (βpad = -0.01; p < 0.05), accounting for 3,8% of the total effect. The increase of 1 SD in the overall environment perception resulted in decrease of 0.09 SD in odds of self-reported hypertension. Also, the increase of 1 SD in leisure-time walking resulted in a decrease of 0.06 SD in odds of self-reported hypertension (Table 4).

**Table 4:** Direct, indirect, and total effects of overall environment perception on leisure walking and self-reported hypertension, in older adults from Florianopolis, Brazil. EpiFloripa Aging Cohort Study, 2017–2019 (n = 1, 292).

Pathways and estimates	Standardized coefficient	Standard error	P value
**Direct Effects** Overall environment perception −> Leisure walking	0.08	0.06	**<0.01**
Overall environment perception −> self-reported hypertension	−0.09		**<0.01**
Leisure walking −> self-reported hypertension	−0.06		**<0.05**
**Indirect Effect** Overall environment perception −> Leisure walking −> self-reported hypertension	−0.01	0.002	**<0.05**
**Total Effect** Overall environment perception**^†^**	−0.21	0.035	**<0.01**

SE: standard error, CI, confidence interval; CFI, comparative fit index; RMSEA, root mean square error of approximation; SEM, standard error of the mean; SRMR, standardized root mean square residual; TLI, Tucker Lewis index. SEM analysis results of the direct, indirect, and total effect of total environment perception on Leisure walking and self-reported hypertension. Adjusted model with sex, age, education level and household income. Model: χ^2^ = 0.178, RMSEA = 0.02, 90 % CI superior = 0.05, probability = 0.97, CFI = 0.93, TLI = 0.91, and SRMR = 0.001. P-value of Structural Equations Model with R.

Note:The total effect reported in the table includes the sum of all pathways in the model, not only the direct and indirect effects of the exposure variable. This approach accounts for the broader structure of relationships within the path model.

## 4 Discussion

The study findings suggest that older adults who perceive better infrastructure for physical activity, increased safety concerning crime and traffic, and an overall positive neighborhood perception are less likely to be diagnosed with hypertension. Furthermore, the association between hypertension and overall environment perception was partially explained by leisure-time walking. These findings highlight the relevance of health-friendly environments for the older adults and suggest specific intervention strategies. Promoting the creation of safe and encouraging spaces for physical activity emerges as an essential measure in the prevention and management of hypertension in this population in Latin American context.

We hypothesize that characteristics of the neighborhood environment may influence the adoption of healthy behaviors and consequently protect against clinical factors. Our findings indicate that older adults who perceived a more supportive overall environment for physical activity are less likely to report a diagnosis of hypertension. This relationship is partially mediated by leisure walking. To our knowledge, no study has explored leisure walking's mediating effects on the link between perceived neighborhood environment and hypertension diagnosis. However, physical exercise was observed to mediate the relationship between greenness, NEWS-based walkability, and hypertension. (Jia et al., 2018). In contrast, objective walkability was associated with reduced odds of hypertension, this effect was not influenced by leisure or transport walking (Adhikari et al., 2021). This disparity might stem from variations in exposure variable measurements. Notably, perceived walkability may hold greater relevance to walking behavior among older adults compared to objective walkability (Kim et al., 2023). In addition to mediation through leisure walking, several other pathways can be hypothesized, such as sedentary behavior, body mass index, and access to health-promoting resources, all of which may contribute to the relationship between the perceived environment and hypertension (Adhikari et al., 2021; Chandrabose et al., 2019a; Jia et al., 2018). However, studies investigating these potential mechanisms remain scarce and inconclusive, making this an under-explored area that warrants further investigation (Chandrabose et al., 2019b).

Given the results, planned interventions in the built environment within walking distance could significantly reduce hypertension among older adults and should be prioritized for managing this chronic condition (Avila-Palencia et al., 2022; Chandrabose et al., 2019b; Koohsari et al., 2020, Lai et al., 2023, Malambo et al., 2016b, de Zorzi et al., 2023). However, longitudinal analyses are needed to further understand the role of physical activity in the relationship between the perceived environment and hypertension.

We found that infrastructure for physical activity is associated with a lower likelihood of reporting a diagnosis of hypertension. Indeed, the presence of these structures correlates with lower systolic and diastolic blood pressure levels (Jones et al., 2021, Li et al., 2009, Savin et al., 2022), reduced likelihood of self-reported hypertension and other cardiovascular diseases in both adults and older adults (Adhikari et al., 2021, Jia et al., 2018, Malambo et al., 2016b). These relationships can be explained because infrastructure characteristics such as walkability, intersection density, pedestrian-friendly features, parks/open spaces and bike lanes promote physical activity, are negatively associated with overweight and obesity, and can improve cardiovascular health (Aziz et al., 2018, Nichani et al., 2020, Sallis et al., 2020, Zhang et al., 2024). The findings also suggest that older adults who perceive heightened security concerning crime and traffic have lower probability of hypertension. This result is corroborated by another middle-income country (Malambo et al., 2016b). Additional studies with adult populations have proposed that increasing crime rates correlate with higher blood pressure and levels of stress (Coulon et al., 2016, Tung et al., 2019). Considering the relationship between stress and higher prevalence of hypertension, it is possible that an increased sense of security reduces stress levels and stimulates physical activity, both of which can decrease the likelihood of hypertension (Grazuleviciene et al., 2021). Moreover, perceived safety measures are negatively associated with BMI, and overweight/obesity related to the outcome (Sallis et al., 2020). Notably, in 2023, the state of Santa Catarina excelled in terms of its high security indices (Ministério da Justiça e Segurança Pública, 2024), which too may explain the protective effect of security found. Enhanced traffic safety correlates with safer environments and reduced levels of noise and pollution, the increase of which has been linked to a higher prevalence of hypertension (Babisch et al., 2014). Moreover, traffic safety may encourage the walking and facilitating access to healthcare services, which can reduce the chance of hypertension (Adhikari et al., 2021, Aziz et al., 2018, Jia et al., 2018). Thus, accumulating evidence suggests that improving infrastructure and safety in both crime and traffic contexts plays a pivotal role in the hypertension in older adults. Public policies that consider the importance of these specific associations can be implemented to promote environments conducive to cardiovascular health in older populations.

No significant associations were found between perceived environmental characteristics and objectively measured elevated blood pressure. Despite research examining various environmental attributes and directly measured systolic and diastolic blood pressure values, substantial associations were not identified (Avila-Palencia et al., 2022, Li et al., 2009, Zanelatto et al., 2019). Comparing our findings with previous studies is difficult due to variations in evaluating SBP and DBP measurements, different measures for environmental attributes, and the inclusion of middle-aged individuals in their samples. One potential explanation for not finding an association between environmental perception and measured blood pressure could be the use of antihypertensive medication, which was not accounted for in this study. Therefore, older adults using medication and maintaining controlled blood pressure might have been categorized as not having the condition, potentially reducing the impact of environmental perception on measured blood pressure. It is emphasized that the health care coverage in Florianópolis is elevate (Voltolini et al., 2023) facilitating the diagnosis, monitoring, and medication control for arterial hypertension (Barroso et al., 2021). Additionally, blood pressure measurements were taken on a single day, incapable of reflecting a diagnosis and subject to daily fluctuations.

Our study has some limitations. The cross-sectional design and observational nature constrain causal inferences. Our measure of hypertension relies on self-report and consequently on access to medical care. Participants who did not report a prior physician diagnosis of hypertension were categorized as not having hypertension in our analyses. This definition may have resulted in underestimating the true prevalence. The use of DAG did not allow the inclusion of the medication variable as a covariate in the main analysis. However, we conducted additional analyses including the medication variable in relation to the objectively measured blood pressure outcome. Since no significant differences were found, we decided not to present these results. Considering the strengths, this study was conducted with a representative sample of older adults in Florianópolis, employing a rigorous methodology that included pilot study, face-to-face interviews, interview quality control, and the use of validated instruments for data collection. Moreover, the article employed both objectively measured blood pressure and participant-reported diagnosis of arterial hypertension, which can help better understand the relationship between neighborhood environmental characteristics and this health outcome. We investigated exposures at the contextual level of the census tract, if individuals living in defined geographic areas with similar socioeconomic status, housing conditions, and available infrastructure share comparable characteristics related to the outcome. Lastly, a DAG was created to illustrate the theoretical model and explain how covariates influence the connection between perceived environmental characteristics and blood pressure, effectively mitigating confounding and selection bias.

## 5 Conclusion

Our research emphasized the significance of neighborhood infrastructure, traffic and crime safety, and overall environmental quality in reducing the likelihood of older adults being diagnosed with hypertension. Therefore, measures to improve urban environments and make communities more conducive to physical activity can play a key role in preventing and controlling hypertension, thus contributing to reducing the global burden of cardiovascular morbidity and mortality. With the increasing prevalence of hypertension, it is imperative that interventions focus not only on clinical treatment but also on promoting cardiovascular-friendly environments. These results support the formulation of comprehensive public health policies targeting both individual risk factors and environmental determinants affecting cardiovascular health among the older adults.

## Declaration of Generative AI and AI-assisted technologies in the writing process

During the preparation of this work the authors used ChatGPT3.5 in order to enhancing readability and language. After using this tool, the authors reviewed and edited the content as needed and take full responsibility for the content of the publication.

## CRediT authorship contribution statement

**Viviane Nogueira de Zorzi:** Writing – review & editing, Writing – original draft, Visualization, Validation, Methodology, Formal analysis, Conceptualization. **Francisco Timbó Neto:** Writing – review & editing, Validation, Conceptualization. **Thamara Hubbler Figueiró:** Writing – review & editing, Validation, Conceptualization. **Danielle de Amaral Macedo:** Writing – review & editing, Validation, Conceptualization. **Lucas Gomes Alves:** Writing – review & editing, Validation, Conceptualization. **Willen Tozetto:** Writing – review & editing, Validation, Conceptualization. **Eleonora d’Orsi:** Validation, Project administration, Methodology, Investigation, Funding acquisition, Conceptualization. **Cassiano Ricardo Rech:** Writing – review & editing, Validation, Supervision, Methodology, Investigation.

## Declaration of competing interest

The authors declare that they have no known competing financial interests or personal relationships that could have appeared to influence the work reported in this paper.

## Figures and Tables

**Supplementary Fig. 1:**
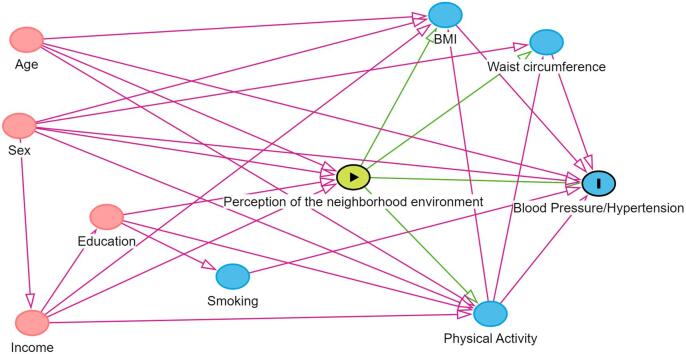


## Data Availability

Data will be made available on request.
