# Supplementary material for: What is the role of leisure-time physical activity in the association between neighborhood environmental characteristics and hypertension in older adults? The EpiFloripa Aging Cohort study
Source: Prev Med Rep. 2024 Oct 18;47:102909. doi: 10.1016/j.pmedr.2024.102909 (PMC11533551; doi:10.1016/j.pmedr.2024.102909)
Supplement: Supplementary Data 2 [file mmc2.docx]

| **Table 2.** Effect of Neighborhood Environment Perception on objectively measured blood pressure in older adults from Florianopolis, Brazil. EpiFloripa Aging Cohort Study, 2017-2019 (n=1,305). | | | | | | | | |  |  |  |
| --- | --- | --- | --- | --- | --- | --- | --- | --- | --- | --- | --- |
| **Variables** | **High Blood Pressure** | | | | | | | |  |  |  |
|  |  | | OR Adjusted  (CI_95%_) | | p-value |  |  |  | |  |  |
| **Land use mix-diversity** |  | |  |  | |  |  | | | |  |
|  | |  | 1 | |  |  |  | | | |  |
|  | |  | 1.10 (0.97-1.25) | | 0.16 |  |  | | | |  |
| AIC | |  | 1599.1 | |  |  |  | | | |  |
| BIC | |  | 1640.4 | |  |  |  | | | |  |
| ICC (%) | |  | 4.6 | |  |  |  | | | |  |
| **Infrastructure for walking/cycling** | |  |  | |  |  |  | | | |  |
|  | |  | 1 | |  |  |  | | | |  |
|  | |  | 0.92 (.81-1.04) | | 0.19 |  |  | | | |  |
| AIC | |  | 1599.4 | |  |  |  | | | |  |
| BIC | |  | 1640.7 | |  |  |  | | | |  |
| ICC (%) | |  | 4.1 | |  |  |  | | | |  |
| **Safety related from traffic** | |  |  | |  |  |  | | | |  |
|  | |  | 1 | |  |  |  | | | |  |
|  | |  | 0.96 (0.85-1.09) | | 0.55 |  |  | | | |  |
| AIC | |  | 1600.7 | |  |  |  | | | |  |
| BIC | |  | 1642.1 | |  |  |  | | | |  |
| ICC (%) | |  | 4.6 | |  |  |  | | | |  |
| **Saffety related from crime** | |  |  | |  |  |  | | | |  |
|  | |  | 1 | |  |  |  | | | |  |
|  | |  | 1.02 (0.90-1.16) | | 0.78 |  |  | | | |  |
| AIC | |  | 1600.9 | |  |  |  | | | |  |
| BIC | |  | 1642.4 | |  |  |  | | | |  |
| ICC (%) | |  | 4.7 | |  |  |  | | | |  |
| **Total Environment Perception- tercile** | |  |  | |  |  |  | | | |  |
| Low | |  | 1 | |  |  |  | | | |  |
| Intermediate | |  | 1.16 (0.87-1.55) | | 0.32 |  |  | | | |  |
| High | |  | 1.08 (0.78-1.50) | | 0.63 |  |  | | | |  |
| AIC | |  | 1602.0 | |  |  |  | | | |  |
| BIC | |  | 1648.6 | |  |  |  | | | |  |
| ICC (%) | |  | 4.9 | |  |  |  | | | |  |
| *AIC: Akaike Information Criterion; BIC: Bayesian Information Criterion; ICC: Intraclass Correlation; 95% CI: 95% Confidence Interval.* *†: Adjusted model with sex, age, education level, household income and antihypertensive medication.*  *Note: Neighborhood Environment Perception variables are presented as z-score.* *The AIC and BIC values were smaller for the adjusted models, indicating a better fit compared to the unadjusted models.* | | | | | | | | |  |  |  |
