# Supplementary material for: What is the role of leisure-time physical activity in the association between neighborhood environmental characteristics and hypertension in older adults? The EpiFloripa Aging Cohort study
Source: Prev Med Rep. 2024 Oct 18;47:102909. doi: 10.1016/j.pmedr.2024.102909 (PMC11533551; doi:10.1016/j.pmedr.2024.102909)
Supplement: Supplementary Data 3 [file mmc3.docx]

Overall environment perception

Adjustment variables

Self-reported hypertension

Leisure walking

0.08*

-0.06*

-0.09*

**Appendix C.** Simplified theoretical model of the direct effects of overall environment perception on leisure walking and self-reported hypertension. The Structural Equation Modeling (SEM) analysis was conducted and presented with standardized coefficients. All models were adjusted for sex, age, education level and household income. ^∗^p < .05.
